# The distribution and impact of common copy-number variation in the genome of the domesticated apple, *Malus* x *domestica* Borkh

**DOI:** 10.1186/s12864-015-2096-x

**Published:** 2015-10-23

**Authors:** James Boocock, David Chagné, Tony R. Merriman, Michael A. Black

**Affiliations:** Department of Biochemistry, University of Otago, Dunedin, New Zealand; The Virtual Institute of Statistical Genetics (VISG), Rotorua, New Zealand; The New Zealand Institute for Plant & Food Research Ltd, Palmerston North, New Zealand

## Abstract

**Background:**

Copy number variation (CNV) is a common feature of eukaryotic genomes, and a growing body of evidence suggests that genes affected by CNV are enriched in processes that are associated with environmental responses. Here we use next generation sequence (NGS) data to detect copy-number variable regions (CNVRs) within the *Malus* x *domestica* genome, as well as to examine their distribution and impact.

**Methods:**

CNVRs were detected using NGS data derived from 30 accessions of *M*. x *domestica* analyzed using the read-depth method, as implemented in the CNVrd2 software. To improve the reliability of our results, we developed a quality control and analysis procedure that involved checking for organelle DNA, not repeat masking, and the determination of CNVR identity using a permutation testing procedure.

**Results:**

Overall, we identified 876 CNVRs, which spanned 3.5 % of the apple genome. To verify that detected CNVRs were not artifacts, we analyzed the B- allele-frequencies (BAF) within a single nucleotide polymorphism (SNP) array dataset derived from a screening of 185 individual apple accessions and found the CNVRs were enriched for SNPs having aberrant BAFs (*P* < 1e-13, Fisher’s Exact test). Putative CNVRs overlapped 845 gene models and were enriched for resistance (R) gene models (*P* < 1e-22, Fisher’s exact test). Of note was a cluster of resistance gene models on chromosome 2 near a region containing multiple major gene loci conferring resistance to apple scab.

**Conclusion:**

We present the first analysis and catalogue of CNVRs in the *M*. x *domestic*a genome. The enrichment of the CNVRs with R gene models and their overlap with gene loci of agricultural significance draw attention to a form of unexplored genetic variation in apple. This research will underpin further investigation of the role that CNV plays within the apple genome.

**Electronic supplementary material:**

The online version of this article (doi:10.1186/s12864-015-2096-x) contains supplementary material, which is available to authorized users.

## Background

The availability of genome sequence data from individuals within a species now enables the investigation of a range of inherited genetic variations at a high resolution. Traditionally, genomic analysis of DNA variants has focused on the identification of single nucleotide polymorphisms (SNPs), and small insertions and deletions (INDELS). However in recent years, other forms of genomic variation have also begun to receive attention. One such form is copy number variation (CNV), defined as a deletion, duplication or insertion of DNA sequence fragments longer than 50 base pairs in length [1].

Studies of CNV in eukaryotic organisms, such as dog [2], barley [3], and human [4], have revealed that 4 to 15 % of a eukaryotic genome is comprised of regions which exhibit variation in copy number between individuals. CNVs have the potential to exert influence on genes by altering both their expression and structure. For example, in humans the common Sotos syndrome generally occurs when a deletion of one copy of the *plasma coagulation 12* (*FXII*) gene exposes a deficiency in the remaining copy [5]. CNV can also contribute to the phenotypic diversity of domesticated animals: in cattle, a partial deletion of the *ED1* gene causes anhidrotic ectodermal dysplasia [6]; and in pigs, white coat color is caused by a duplication involving the *KIT* gene [7, 8]. CNV distribution is not random across genomes. As an example, segmental duplications (SDs), which are sections of DNA with near-identical sequence, are considered hotspots for CNV formation [9].

CNV is a common feature of plant genomes, and recent work has highlighted its functional relevance. In barley, genes affected by CNV were enriched for potential functions in disease resistance [3], a finding that is consistent with the results of CNV studies in soybean [10] and maize [11]. In wheat, CNVs in *Vrn-A1* and *Ppd-B1* have been shown to influence flowering time [12], and a CNV at the *Rht-D1b* locus is associated with dwarfism [13]. In soybean, increased copy-number of an allele of the *Rhg1* gene is responsible for nematode resistance [14], while in barley, boron tolerance is associated with a CNV at the *Bot1* locus [15]. Based on these reports, there is evidence that CNVs are frequently associated with the biotic and abiotic stress response in annual crop species. However, no analysis of CNVs in perennial tree species has been performed to date. We hypothesize that CNVs are particularly relevant as a source of genetic variability that can be rapidly utilized for adaptation to stress in long-lived plants, including horticultural and forest tree species.

The domesticated apple (*Malus* x *domestica* Borkh*.*) first appeared in the Near East around 4,000 years ago. In 2010, a high-quality draft genome of ‘Golden Delicious’ was released [16]. Pairwise comparison of the assembled genome revealed that a whole genome duplication (WGD) had recently <50 million years ago occurred in the Pyrinae (which includes fruit species such as apple, pear and quince), leading to an almost doubling of chromosomal number. However, non-global duplication and deletion events, which are localized to individual loci or regions and can thus be considered to represent CNV, have not previously been described in the apple genome. Apple researchers have used Next-Generation Sequencing (NGS) technologies to detect SNPs across the genome, enabling the development of apple SNP arrays used for genomic selection in apple breeding programs and for fine trait mapping [17–19]. NGS-based re-sequencing in the apple genome has indicated that domesticated apple is significantly heterozygous, with 4.8 SNPs per kb, and a recent study has revealed that multiple introgression events with wild apple species such as *Malus sylvestris* and *M. sieversii* have shaped the genomes of modern domesticated cultivars [20].

Hybridization-based methods, such as array comparative genomic hybridization (aCGH), were the first high-throughput approaches used to identify CNVs at the genome-wide scale [21]. More recently however, NGS technologies combined with new analytical approaches, such as read-depth, paired-end, and split-reads analysis, have become popular [22]. Analysis of read-depth is an effective method for CNV detection, relying on detection of changes in the depth of coverage across the genome as being indicative of changes in the underlying copy-number [23]. The aim of our study was to detect CNV regions (CNVRs), in the apple genome using low-coverage (1x to 5x) NGS re-sequencing data from 30 apple varieties grown or used for cultivar breeding worldwide.

## Methods

### Plant material and next-generation sequencing

A low-coverage NGS dataset for 30 domesticated apple (*M.* x *domestica*) accessions was developed using Illumina GAII technology, with one lane per individual as described in [19]. These varieties represent founders, intermediate ancestors, or important breeding parents used extensively in apple breeding programs worldwide. The complete set maximized coverage of the genetic background of the world’s domesticated apple. Individuals in the set were *Malus* x *domestica* ‘Braeburn’, ‘CrimsonCrisp’, ‘Delicious’, (both the original form and the derived mutant ‘Red Delicious’), ‘Duchess of Oldenburg’, F2268292-2, ‘Geneva’, ‘Golden Delicious’, ‘Granny Smith’, ‘Haralson’, ‘Honeycrisp’, ‘Idared’, ‘James Grieve’, ‘Jonathan’, ‘McIntosh’, ‘Ralls Janet’, ‘Red Dougherty’, ‘Rome Beauty’, ‘Splendour’, ‘Big Red’, ‘Papa Noel’, ‘Merton Russet’, ‘Wilmont Russet’, ‘David’ o.p., ‘Malling 9’, and four advanced selections. The raw sequencing data for many of the accessions is publicly available, and for the other accessions data can be made available on request (Additional file 1).

### Sequence alignment and CNVR identification

The reads from the 30 *Malus* x *domestica* samples were aligned to the pseudohaplotype assembly of ‘Golden Delicious’ [16] using the Burrow’s wheeler aligner (BWA v 0.7.5a) maximal exact matches (MEM) command [24, 25]. To reduce the impact of highly abundant organelle DNA on the read depth analysis, a LAST (v 6.21) [26] database was created using apple mitochondrial DNA [27] and apple chloroplast DNA [28]. Sections of the assembly with a highly confident match (score > = 40) were considered to be derived from organelles. Additionally, the locations of repeat sequences, such as retrotransposons, were obtained [29] and reads that overlapped repeat sequences, mitochondrial, or chloroplast locations by at least one base pair were removed using bedtools (v2.19.1) [30]. As duplications can occur during the PCR amplification process and create spurious spikes in read-depth signal, these were identified and removed using Picard’s (v 1.124) MarkDuplicates functionality [31].

The binary alignment format (BAM) files used for CNV analysis were filtered based on mapping quality (reads with MAPQ < 20 were discarded), and processed using CNVrd2 [32] (v1.4.0), where the read-depth was counted in 3000-bp non-overlapping windows. Following this, windows that covered a segment of the assembly with greater than 50 % Ns were removed. The GC-content of a genomic region influences the read-depth when using Illumina sequencing [33]. This presents a challenge for read-depth analysis and was minimized using the GC-content adjustment method that is implemented in CNVrd2. The resulting GC-adjusted read count data were then standardized within and between samples using CNVrd2. As a result of standardizing across multiple samples, the influence of mappability bias, which is present at some complex regions, was minimized. Coverage statistics were generated using the R programming language (v3.1.1) [34] with figures created in R using the ggplot2 (v1.0.0) [35] and gplots (v2.16) [36] packages. CNVrd2 uses the DNACopy package [37] from the Bioconductor project [38], which employs a binary segmentation algorithm to segment the normalized read-counts. This procedure results in a segmentation score for each window in each sample. These scores represent significant changes in read-depth, with numbers greater than zero indicating a change to increased read-depth, and numbers less than zero indicating a change to decreased read-depth. These changes in read-depth are indicative of changes in the underlying copy-number within a sample. Although these raw segmentation scores can be processed to obtain integer copy-numbers, for this work we chose to analyze the raw segmentation scores, focusing on detecting regions that varied significantly in segmentation score between the apple accessions. Spearman’s correlation was used to investigate the relationship between read-depth and number of segmented regions. A visualization of the distribution of CNVRs throughout the apple was created using Circos (v0.67-4) [39]. In addition to detecting CNVRs with CNVRd2, an independent analysis was performed for comparison using the filtered BAM files with the R package cn.MOPS [40].

To identify CNVRs, a trimmed standard deviation (removing the min. and max. values) was calculated to compare the segmentation scores in each sample for each window. This trimmed standard deviation was used to remove outliers from the calculations. Permutation testing (10,000 permutations per chromosome) was used to determine CNVR significance. This involved shuffling the segmented regions in each sample, recalculating trimmed standard deviations, and calculating an empirical false discovery rate (FDR) [41]. The FDR was calculated at different thresholds, with the genome-wide FDR calculated as a mean of each chromosomal value weighted by chromosome length. A genome-wide trimmed standard deviation threshold of 0.25 was used to determine whether a window was within a CNVR, and neighboring windows above this threshold were merged to form the CNVRs.

### SNP Array support for candidate CNVRs

A SNP dataset was used for validation of CNVRs which contained information on the scoring of 10,685 SNP polymorphic markers from the apple Illumina Infinium 20 k SNP array [18] screened over 185 apple accessions from the Plant & Food Research germplasm collection that have a similar genetic background to the varieties used for read-depth analysis. As the 20 k SNP array data comes from an independent experiment that utilized microarray technology, it presented us with an opportunity to strengthen the evidence that the CNVRs detected with read-depth from sequencing data were reliable. SNP genotyping assumes diploid copy number, an assumption that is violated when a SNP falls within CNVRs, as genotypes such as AAB, carrying an extra copy of the “A” allele, and AØ, missing a copy of the “B” allele, may be present [42]. The B allele frequency (BAF), which is the proportion of signal explained by the B allele, has an expected value for heterozygotes of 0.5, and for homozygotes of 0 or 1. Odd numbers of alleles at a locus, such as AAB, may give values falling outside these values. The B allele frequency (BAF) was extracted for each SNP data point using the Illumina GenomeStudio software. CNVRs were tested for enrichment of SNP markers with a B allele frequency (BAF) between 0.05 and 0.35, or 0.65 and 0.95, in at least 10 % (19) of samples. Fisher’s exact test [43] was used to check whether the array design was biased against SNPs being located in CNVRs.

### Gene model annotation and functional analysis

The consensus gene models were obtained from the Genome Database for Rosaceae [44]. A gene model was considered to overlap a CNVR if more than 70 % of its bases were within the boundaries of a CNVR. Gene Ontology analysis [45] was performed using the Fisher’s exact test. The resulting *P*-values were adjusted using the FDR-controlling approach of Benjamimi and Hochberg [41]. A separate enrichment test was performed using predicted resistance gene models obtained from the supplementary information of the publication describing the apple genome [16]. The repeat sequences were classified further by BLAST searching [46] against the RepBase19.12 [47] database. The flanking regions of CNVRs were determined by creating a list of positions 10 kb either side of each CNVR, and merging the overlapping regions. The number of elements for each class that entirely overlapped or did not overlap was calculated, followed by enrichment testing using a Fisher’s exact test. Genic regions were determined by creating a list of positions 10 kb either side of each gene model. The average GC content of the flanking regions of both the CNVRs and the genic regions was calculated on a per chromosome basis.

## Results

### Data summary and CNVR identification

Before quality control (QC), the average sequencing coverage for each variety varied between 3.51× and 13.60×. After QC, which included removing reads overlapping the organelle and repeated regions, the average sequencing coverage for each variety was reduced to between 0.95× and 4.96× (Additional file 1), indicating that between 63 and 73 % of the reads mapped to the excluded regions.

**Fig. 1:**
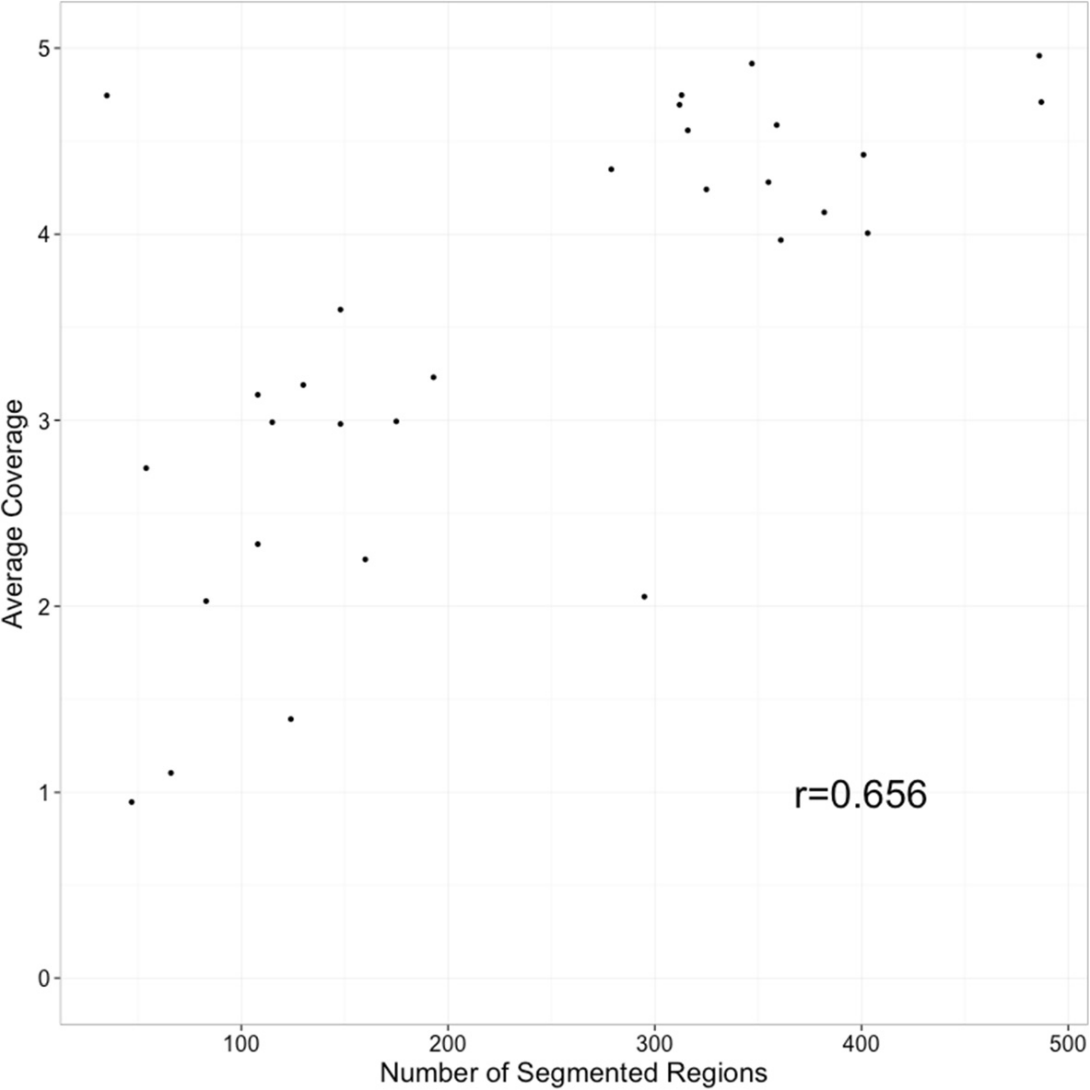
Relationship between number of segmented regions detected in each apple accession and the apple genome coverage. Average coverage per apple accession versus total number of segmented regions, determined using CNVrd2

**Fig. 2:**
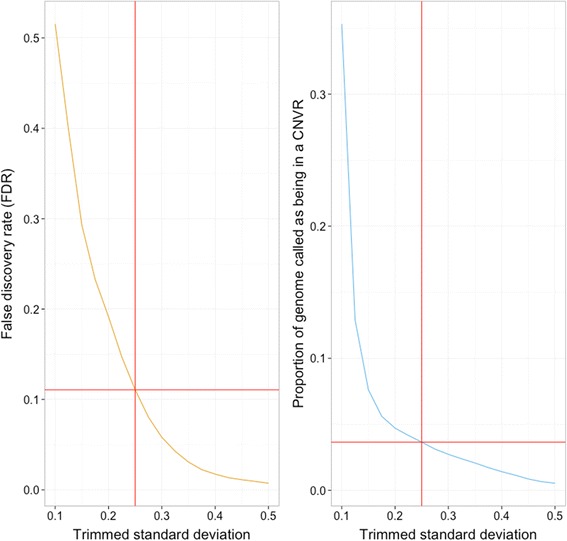
Relationship between the false discovery rate (FDR) estimated using permutation analysis, and the proportion of the genome comprising copy-number variable regions (CNVRs) in the apple genome, as a function of significance threshold. The red lines intersect at a threshold of 0.25, which was the threshold used for downstream CNV analysis using re-sequencing data of 30 apple accessions

The read-depth CNV detection method is based on an assumption that the number of reads originating from a region of a genome after removing technical bias is indicative of the copy number for that region. Reads were counted in 3-kb non-overlapping windows. Prior to normalization the median read-depth for all windows was 38. Following GC-content adjustment the median read-depth became 16.8, this highlights the impact that GC content can have on read-depth. After normalization, these windowed read counts were segmented into regions with similar signal values. Investigation of the relationship between coverage and the number of segmented regions revealed a positive correlation (Spearman’s correlation of 0.656) between coverage and the number of regions detected, which is an indication that CNVs are more likely to be detected in samples of higher coverage (Fig. 1). We used a linear model to further quantify this relationship, regressing the number of segmented regions against sequencing coverage. The beta estimate from this model was 82.58 (*P* < 1e-5, *T*-test), meaning that for every unit increase in average coverage; the model estimated that the average number of segmented regions increased by 82.58. Although integer copy number assignment for an individual sample can be performed using the read-depth method, given the low-coverage sequencing data used in our study and the incomplete apple genome assembly, we chose instead to focus on CNVRs that displayed significant variation in segmentation scores and not to attempt integer copy number assignment. These significantly variable CNVRs were determined by summarizing each window using a trimmed standard deviation (removing the minimum and maximum), followed by a permutation testing procedure to calculate the threshold used to identify potential CNVRs: any window with a trimmed standard deviation above this threshold was considered to lie within a CNVR. A threshold of 0.25 gave an acceptable FDR of 11 %, and was used in downstream analyses (Fig. 2).

**Fig. 3:**
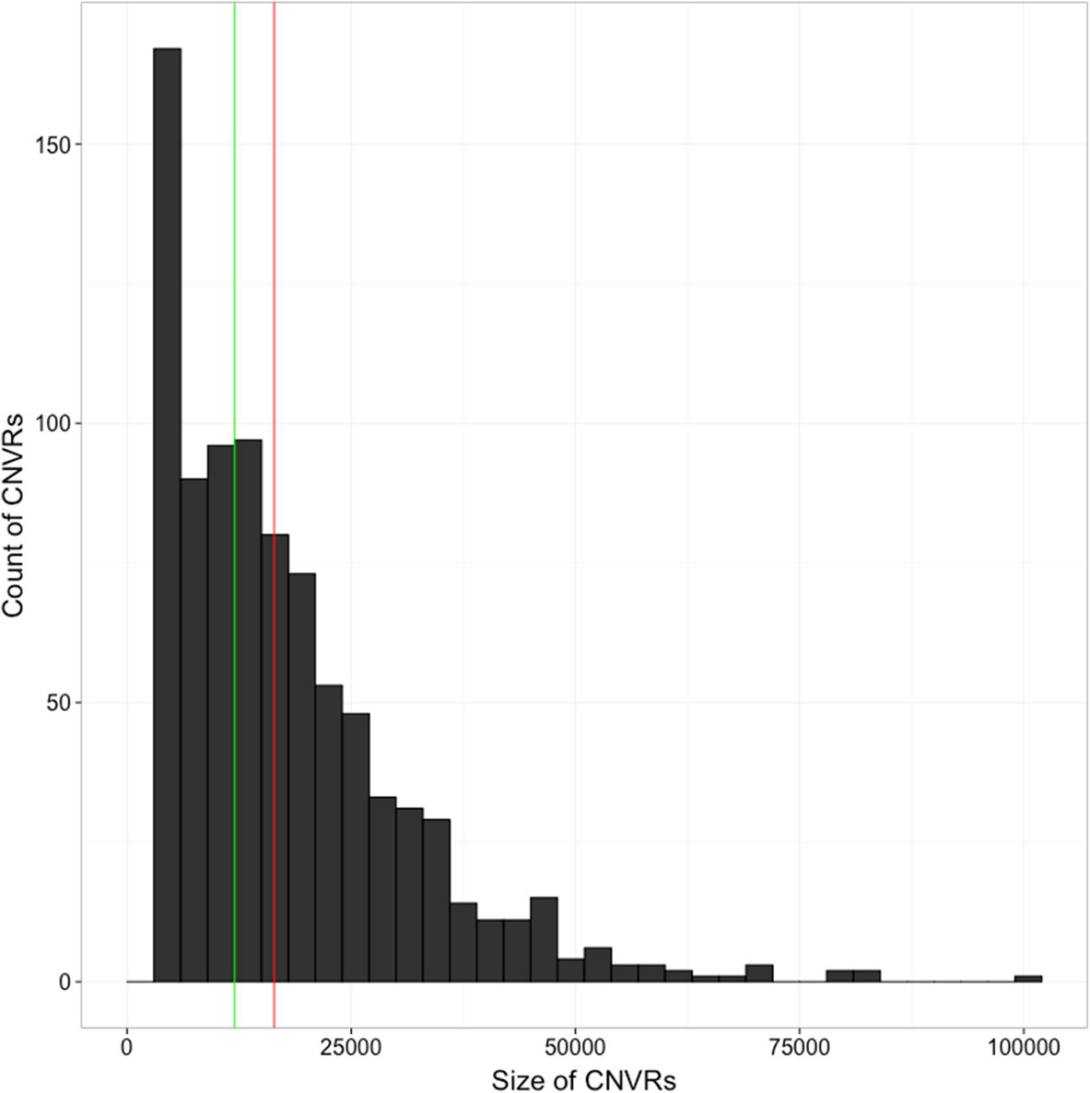
Distribution of length and frequency of copy-number variable regions (CNVRs) in the apple genome. Size (in base pairs) versus number of CNVRs. The green and red lines represent the median and mean CNVR size, respectively

**Fig. 4:**
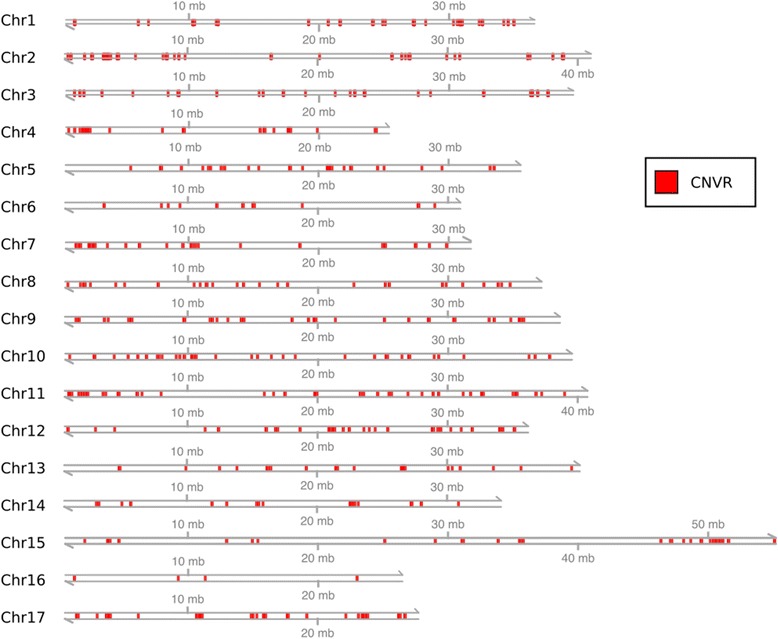
Copy-number variable regions (CNVRs) in the apple genome. The 17 grey lines represent all the chromosomes of the ‘Golden Delicious’ v1.0p pseudohaplotype assembly [16]. Red sections indicate the locations of the 876 CNVRs. mb: megabases

The 876 CNVRs detected using the above threshold spanned a total of 14.4 Mb or 3.5 % of the ‘Golden Delicious’ v1.0p pseudohaplotype assembly (Additional file 2). They ranged in size from 3 kb to 99 kb, with an average length of 16.4 kb, and a median length of 12 kb (Fig. 3). CNVRs appeared to be non-randomly distributed throughout the genome (Fig. 4). The percentage of an individual chromosome in CNVRs varied between 1.5 % for chromosome 16 and 5.7 % for chromosome 10 (Additional file 3). When the removal of regions split a large CNVR, it was considered as two smaller CNVRs. In addition to the read-depth analysis performed using CNVRd2, CNVRs were detected with the R package cn.Mops (data not shown). These CNVRs based on cn.Mops analysis spanned a total of 41.8 Mb or 10 % of the apple genome. In total, 59.6 % of the exact bases in the CNVRs were detected both by CNVRd2 and cn.Mops. If one relaxes the criteria and counts the entire CNV length reported from CNVRd2 when an overlap is observed, then 87.5 % of the CNVR bases were recovered.

### Repeat analysis and GC content

Repeated sequences were investigated for their relationship with CNVRs. A list of 10-kb regions flanking CNVRs was developed, with overlapping sections merged. The repeated elements that are the most numerous in the apple genome (*Copia*, *Gypsy*, *hAT*, *Cassandra*, and LINE) [16] were tested for enrichment within the flanking regions. A significant depletion was observed for *Gypsy* elements (*P* = 0.007, Fisher’s exact test) and a significant enrichment for *Copia* elements (*P* = 0.006, Fisher’s exact test) (Table 1). No significant enrichments or depletions (*P* > 0.05, Fisher’s exact test) were observed for *hAT*, Cassandra, and LINE elements, and no overall enrichment (*P* > 0.05, Fisher’s exact test) was detected for repeated elements. The genome-wide GC content of CNVRs was nominally (*P* = 0.03, *T*-test) higher (average 37.9 %) than that of the pseudohaplotype assembly (37.8 %). In contrast, the genome-wide average for genic GC content was nominally (*P* = 0.02, *T*-test) lower than that of CNVRs (average 37.6 %) (Additional file 3).

**Table 1:** Number of repetitive elements contained in the 10 kb of sequence up- and downstream of the copy-number variable regions (CNVRs) in the apple genome

CNVR flanking Regions			Non-CNV flanking regions		
Repeat type	Observed	Expected	Observed	Expected	*P*-value
*Gypsy*	1917	2087.5	85,346	85175.5	0.007
*Copia*	815	707.7	28,767	28874.3	0.006
*Cassandra*	245	225.2	9169	9188.8	0.375
*LINE/RTE*	264	251.5	10,250	10262.5	0.624
*hAT*	402	350.6	14,253	14304.4	0.065
Total	3643	3622.4	147,785	147805.6	0.812

### Independent experimental support for candidate CNVRs using a SNP array dataset

**Fig. 5:**
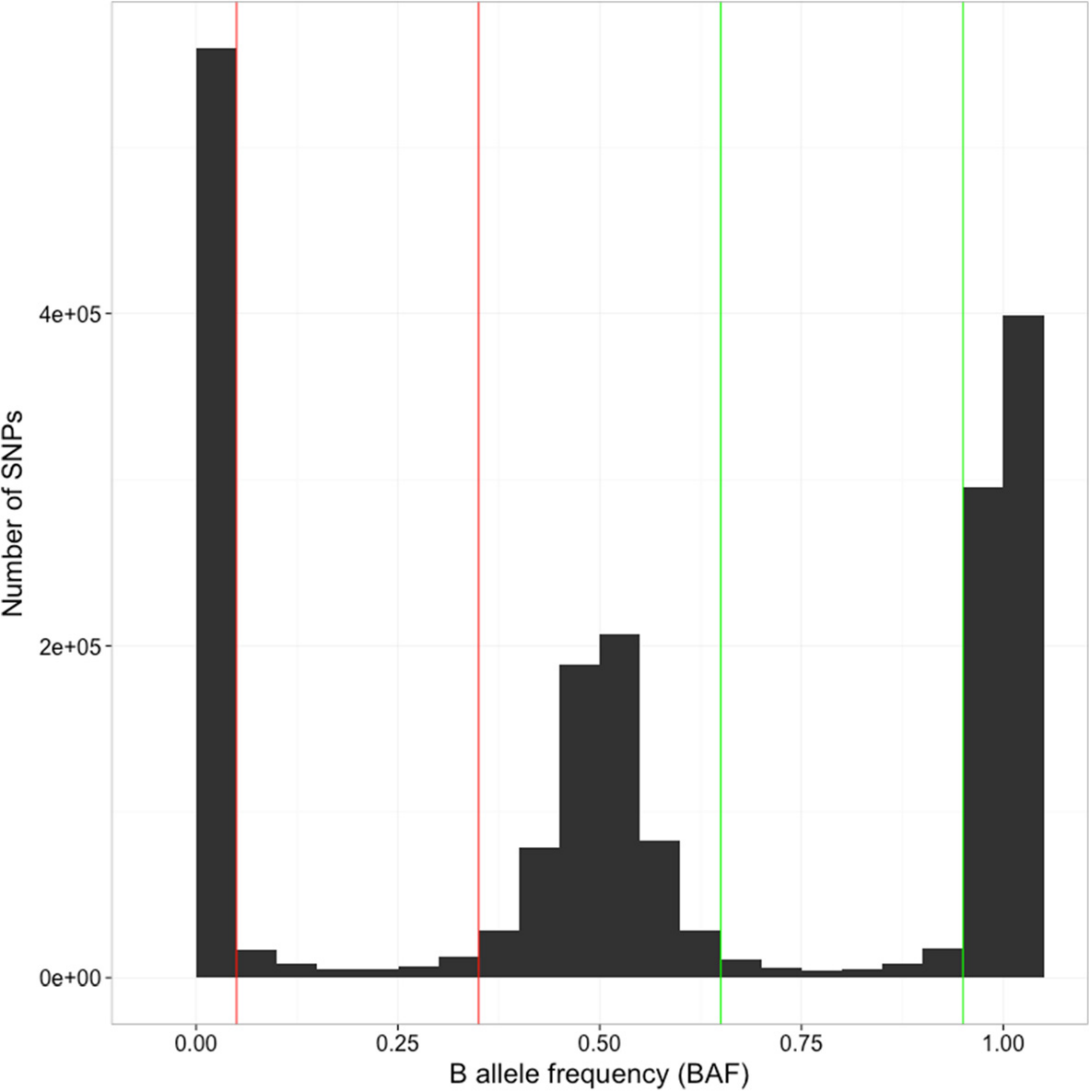
Distribution of B allele frequencies for the 20 k SNP array screened on 185 apple accessions. The count of all B allele frequencies (BAFs) is displayed in windows of size 0.05 from a dataset containing 185 apple accessions genotyped on the apple Infinium 20 k SNP array [18]. The ranges 0.05–0.35 and 0.65–0.95, shown by red and green lines respectively, indicate the ranges where an individual BAF was considered aberrant

Although low density SNP microarrays do not enable copy-number detection directly, because of their limited probe density, they do present a method of independently validating CNVRs detected by read-depth analysis. We accomplished this by extracting the BAFs from an apple Illumina Infinium 20-k SNP array dataset that contained the genotyping information for 185 accessions. After the removal of SNPs that overlapped the windows which were previously removed from the analysis because they contained Ns, repeats, or organelle DNA, 10,685 SNPs remained. BAF thresholds of 0.05–0.35 and 0.65–0.95 were considered aberrant (Fig. 5) and when 19 (10 %) of samples fell within these ranges, the SNPs were considered as having an aberrant BAF pattern indicative of CNV. CNVRs contained 49 of the 1845 (2.6 %) SNPs with aberrant BAFs, and 115 of the 8840 (1.3 %) normal (disomic) SNPs, representing an approximately two-fold enrichment of SNPs with aberrant BAFs, within CNVRs (*P* < 0.0001, Fisher’s Exact test). Interestingly, 98.5 % of the SNPs represented on the apple Illumina Infinium 20 k SNP array were located in non-CNV regions, indicating a significant depletion of SNPs in CNVRs (*P* < 1e-13, Fisher’s Exact test) and suggests that the SNP array design was biased away from CNVRs.

### Functional analysis of CNVRs

Of the consensus gene models from the Genome Database for Rosaceae, 29,015 were located in genomic regions that were analyzed. A total of 845 (2.9 %) of these gene models exhibited a minimum of 70 % of their base pairs overlapping putative CNVRs (Additional file 4). Significant depletion of gene models within CNVRs was observed (*P* < 1e-6, Fisher’s Exact test), with the proportion of the genome assembly spanned by CNVRs (3.5 %) being greater than the proportion containing gene models. Over half (470) the CNVRs did not contain a gene model, with the remaining 406 regions (46.3 %) containing at least one gene model. Functional enrichment analysis of CNVRs revealed 20 GO terms overrepresented after FDR *p*-value adjustment (Table 2). These terms included “apoptotic process”, “innate immune response” and “defense response”, for which a significant proportion of annotations were found to originate from resistance (R) gene models. This class of genes are proteins containing nucleotide-binding sites (NBS or NBC-ARC domains) and C-terminal leucine-rich repeats (LRR), and are key components of the immune response in plants [48, 49]. To investigate the relationship between R gene models and CNVRs directly, we obtained a list of 992 resistance (R) gene models from the apple genome publication [16]. Of the 992 R gene models, 268 were located in the GD pseudohaplotype assembly we used for CNV detection, and 47 (16.3 %) of these R gene models overlapped CNVRs (Additional file 5). Furthermore, R gene models were enriched greater than five-fold within CNVRs (5.5 % of total gene models) compared with outside these regions, where they comprised 0.8 % of total gene models (*P* < 1e-22, Fisher’s exact test) (Table 3). This significant enrichment of R genes is consistent with results from the cn.MOPS analysis (*P* < 1e-47 for cn.MOPS results only; *P* < 1e-19 for the intersection between cn.MOPS and CNVrd2). Ten of these enriched GO terms were not attributable to R gene models, such as “ion transport”, “chloride transport”, and “voltage-gated chloride channel activity”.

**Table 2:** Significant Gene Ontology terms (*P* < 0.05 after False-discovery rate correction) for gene models located within copy-number variable regions (CNVRs) in the apple genome

Geno Ontology ID	Description	*P* value
GO:0006915	apoptotic process	4.23E–48
GO:0031224	intrinsic component of membrane	4.09E–43
GO:0004888	transmembrane signaling receptor activity	1.16E–42
GO:0045087	innate immune response	2.75E–42
GO:0005515	protein binding	1.03E–32
GO:0007165	signal transduction	8.93E–31
GO:0005524	ATP binding	9.26E–22
GO:0006952	defense response	2.40E–21
GO:0005216	ion channel activity	0.006
GO:0006508	proteolysis	0.009
GO:0004674	protein serine/threonine kinase activity	0.011
GO:0006468	protein phosphorylation	0.011
GO:0043086	negative regulation of catalytic activity	0.011
GO:0005516	calmodulin binding	0.012
GO:0005247	voltage–gated chloride channel activity	0.026
GO:0042802	identical protein binding	0.028
GO:0004497	monooxygenase activity	0.042
GO:0006821	chloride transport	0.042
GO:0030552	cAMP binding	0.045
GO:0016710	trans-cinnamate 4-monooxygenase activity	0.049

**Table 3:** Resistance (R) gene models located within copy-number variable regions (CNVRs) in the apple genome

MPIDs	Chromosome	Start (bp)	End (bp)	Width (bp)
MDP0000816743	chr1	24886957	24890824	3867
MDP0000144874	chr1	30626140	30628752	2612
MDP0000634037	chr1	30801610	30806248	4638
MDP0000257201	chr1	30817742	30823513	5771
MDP0000282740	chr1	30901618	30906893	5275
MDP0000149577	chr1	31011253	31015489	4236
MDP0000685087	chr2	2694102	2698622	4520
MDP0000483490	chr2	2705247	2706068	821
MDP0000577795	chr2	3531631	3532194	563
MDP0000478441	chr2	3606656	3610584	3928
MDP0000522693	chr2	3615408	3616082	674
MDP0000563856	chr2	3870168	3872449	2281
MDP0000466397	chr2	3940831	3943960	3129
MDP0000458576	chr2	3967880	3972931	5051
MDP0000746482	chr2	3985869	3990107	4238
MDP0000460947	chr2	4615319	4619759	4440
MDP0000512349	chr2	4701149	4701745	596
MDP0000573272	chr2	4731508	4735373	3865
MDP0000206582	chr2	9335438	9338647	3209
MDP0000301892	chr2	9357524	9362451	4927
MDP0000640508	chr2	38880821	38885458	4637
MDP0000261720	chr2	38910867	38912498	1631
MDP0000303694	chr3	22748766	22753650	4884
MDP0000173893	chr4	15497478	15501779	4301
MDP0000399716	chr6	15009829	15020552	10723
MDP0000148432	chr6	15038138	15040970	2832
MDP0000257606	chr6	15042847	15050886	8039
MDP0000166759	chr7	1337480	1340290	2810
MDP0000196621	chr7	1370821	1373441	2620
MDP0000259862	chr7	1591348	1594205	2857
MDP0000493661	chr7	2442347	2446032	3685
MDP0000321189	chr7	2458873	2466023	7150
MDP0000685425	chr8	11405302	11410962	5660
MDP0000586712	chr9	1526915	1531337	4422
MDP0000125338	chr9	19770884	19773602	2718
MDP0000472864	chr10	892484	896484	4000
MDP0000264448	chr10	10432552	10452135	19583
MDP0000193087	chr11	1928588	1932682	4094
MDP0000732188	chr11	2110384	2116572	6188
MDP0000212932	chr11	3506106	3509841	3735
MDP0000230932	chr11	3712888	3720930	8042
MDP0000171723	chr13	21556571	21559228	2657
MDP0000168665	chr14	13071029	13075189	4160
MDP0000272830	chr15	15393697	15404409	10712
MDP0000680657	chr15	48113339	48116942	3603
MDP0000312626	chr17	1585061	1588664	3603
MDP0000291677	chr17	17704304	17710114	5810

## Discussion

Characterization of the apple genome has focused primarily to date on whole genome duplication, the detection of SNPs, the prediction of genes and the characterization of gene families, as well as generation of an inventory of repeated elements. Structural variations, such as copy-number, have not been studied in detail, in apple or indeed in other fruit tree species. In short-lived crop species, such as maize and rice, copy-number variation has been investigated using NGS and microarray technologies, and a growing consensus has emerged as to the agricultural relevance of CNV [52]. In particular, genes located in CNVRs have been observed to be enriched for stress-related response genes, including the canonical R-genes, which contain a leucine rich-repeat (LRR) domain. This highlights the importance of CNV in relation to agricultural crop genetics, and the need to characterize this type of variation in horticultural species. In this study, we used NGS data from 30 apple accessions to identify 876 copy-number variable regions (CNVRs). The CNVRs within the apple genome overlap 845 gene models and are enriched for R gene models. A significant enrichment of SNPs with aberrant BAFs was observed within CNVRs by using a SNP derived from a screening of 185 individual apple accessions with similar genetic background to those of the 30 varieties used for read-depth analysis. This strengthens the evidence that the regions we detected represent true CNVRs.

Although CNV had not been investigated previously in the apple genome, there has been some evidence that CNV might play an important biological role here. A study investigating QTLs associated with bud phenology in apple identified a candidate *E3 ubiquitin protein* encoding gene present as a tandem array of 14 copies in ‘Golden Delicious’, making it a putative CNV [50]. More recently, a study that was performed to design SNP markers for eight major disease resistance loci, encountered two problems that can be explained by the occurrence of CNV in these regions [51]. Firstly, for the *Rvi2*, *Rvi4* and *Rvi11* loci conferring resistance to apple scab (*Venturia inaequalis*), the presence of paralogs made it difficult to design primers for the SNPs linked to the respective loci, which amplified alleles only at the specific region of interest, without co-amplifying the paralogous sequences. Paralogs located in tandem would be considered a segmental duplication, which are hotspots for CNVs [9]. Secondly, SNPs linked to *Pl2*, a major locus conferring resistance to powdery mildew (*Posdosphaera leucotricha*), failed to amplify when resistance was not observed. This would be expected if resistance were conferred by the insertion of a segment of DNA, which contains the SNPs that failed to amplify, and is absent from susceptible individuals [51].

### Data QC and analysis approach

Because of apple’s high heterozygosity, its genome was not assembled as a single reference sequence but rather into four haplotypes, or individual sequences. The primary pseudohaplotype assembly (*Malus* x *domestica* Whole Genome v1.0p) [53], which was used in this analysis, represents the shortest path through the sequence scaffolds. This reference was used to facilitate short-read mapping and read-depth analysis, using a workflow analogous to studies in diploid mammals, for which many bioinformatic tools have been developed [22]. Repeated elements, which are estimated to cover 42.4 % of the apple genome [16], create a problem for read-depth analysis, as reads which map to repeat regions of the reference create noise in the read-depth signal. This interferes with data normalization and subsequently the downstream segmentation procedure. Many CNV studies attempt to correct for this effect by masking repeat regions before alignment (i.e., changing the position in the reference to an ‘N’) [54, 55]. However, as the quality score of reads is determined by the uniqueness of the mapping position, masking may lead to reads that originate from the repeated sequences mapping spuriously to other locations, with potentially high quality scores. In our analysis, reads were first mapped, and only then were repeated elements removed. This represents an alternative and potentially improved approach to overcoming the problem of repeated sequences. The presence of chloroplast and mitochondrial DNA within the reference genome creates a similar issue, as the large number of these organelles in each cell would lead to large numbers of reads mapping to these regions, potentially interfering with the read-depth approach. In the analysis presented here, reads mapping to organelle-derived regions were removed before CNV segmentation.

Low-coverage NGS re-sequencing data were used to identify the CNVRs that exhibited significant variation in copy-number within a set of apple accessions with genetic links to international breeding programs. The CNVRs identified were found to vary greatly in copy-number among the accessions, and represent candidate gene loci that may be involved in control of variability in traits, such as pest and disease resistance. The number of samples used (30 apple accessions) is higher than employed in some recent read-depth studies in domesticated organisms [e.g., the number of samples was five and 16 in CNV studies of cows [55] and pigs, respectively [54]]; however, is smaller than studies in maize [56] and soybean [10], which involved 278 and 302 samples, respectively. The average coverage for sequencing of our samples ranged from 0.95× to 4.96×, which is relatively low and might have introduced noise into the read-depth signal. Our finding that a strong correlation was observed in our dataset between sequencing coverage and the number of detected CNVs suggested that the read-depth method is more efficient at detecting copy-number changes in higher-depth samples, and as low-coverage data do not enable reliable calling of exact copy number at specific loci for individuals, we opted to analyze the variation among segmentation scores across the 30 varieties to provide an inventory of CNVRs in the apple genome. A trimmed standard deviation was chosen (removal of the min. and max. values), to reduce the impact of outliers potentially driven by noise, and a permutation test was used to assess the likelihood of finding such a variable region by chance. This allowed us to determine a conservative threshold for CNVR identity, with what we considered an acceptable FDR of 11 % [41].

### Patterns of CNVRs in the apple genome

The read-depth analysis identified 876 CNVRs in the apple genome, ranging in length between 3 kb and 99 kb, with an average of 18 kb. It should be noted that because the CNVRs separated by windows that were removed were not merged, the number of detected CNVRs is likely to be an overestimate, as a number of CNVRs interleaved with removed regions may actually comprise a single large CNVR. Our results suggest that 3.5 % (14.4 Mb) of the analyzed assembly lies within a CNVR. This percentage is lower than found in previous CNV studies in plants; for example, the CNV percentages found in maize [56] and barley [3] were 10 and 14.9 %, respectively. This discrepancy might be explained by the lower degree of polymorphism observed among the apple accessions employed in our study compared with that in maize lines, whereby apple has one SNP every 288 bp on average [19], versus every 60 bp in maize [57]. However, it is possible that we might have underestimated the total contribution of CNVs to the apple genome because of the low-coverage sequencing data, strict quality control, and our analysis approach, which focused on detecting only CNVRs that exhibited high variation among the samples.

### In the apple genome CNVRs are enriched in resistance genes

In total, 845 gene models overlapped the CNVRs detected. Initial functional enrichment analysis suggests that these gene models are involved in ion transport, signal transduction, and the defense response. This result is concordant with other studies in a wide range of species, such as humans [4], barley [3], and soybean [10], which have noted that immunity-related genes are frequently found within CNV regions. Fast-mutating CNVs have been recognized as an evolutionary driving force for organisms to adapt to changes in environmental conditions and the introduction of new pests and diseases [58]. This phenomenon is particularly important for long-lived tree species, which have a considerably lengthier generation time than their pathogens and which cannot migrate large distances to adapt to new conditions. In light of this, we propose that CNV is an extremely important adaptive genetic mechanism in tree species, even more so than for other organisms.

### Co-location between CNVRs and trait loci in apple

**Fig. 6:**
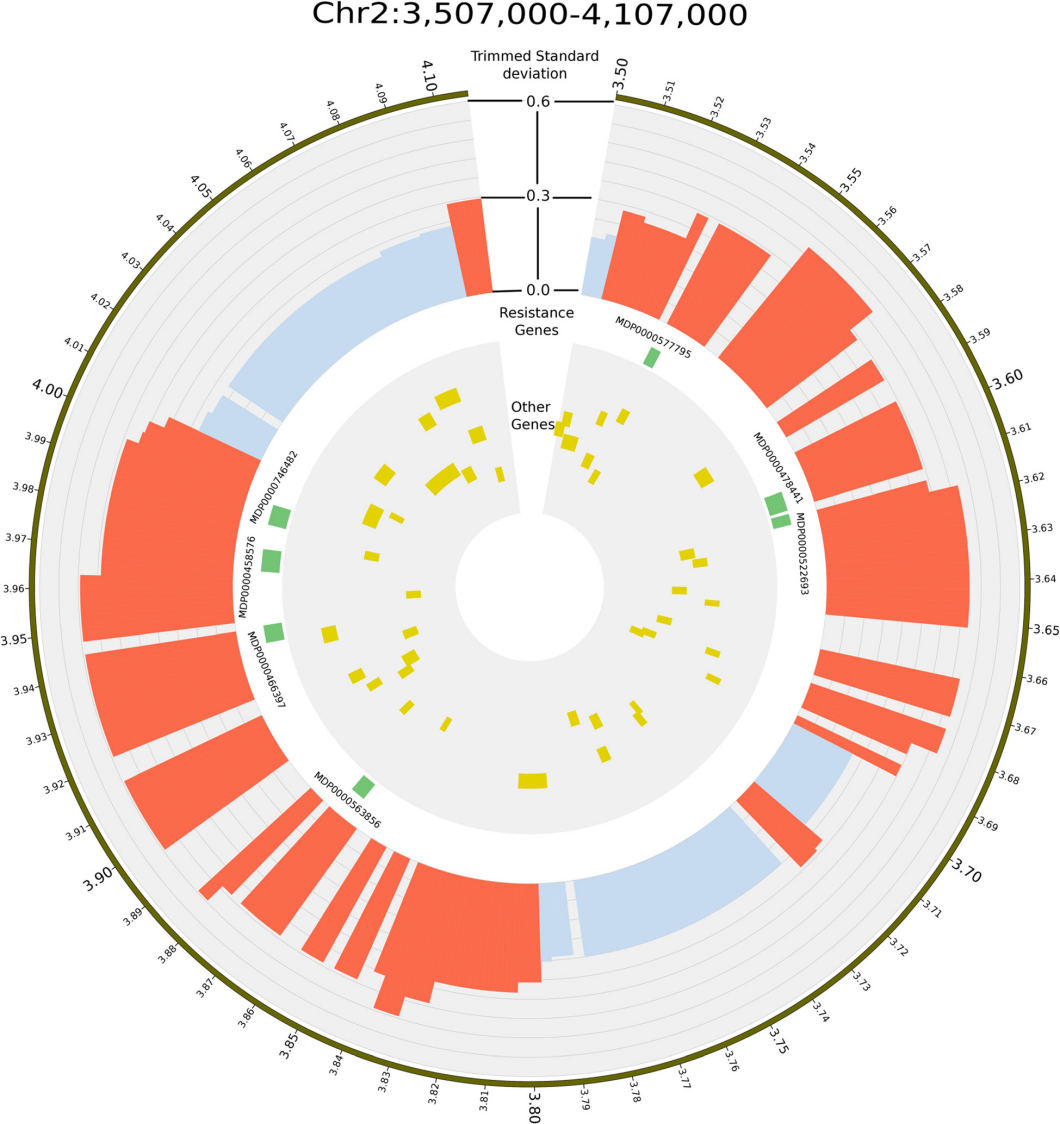
Circos plot displaying the copy-number variable regions (CNVRs), R genes, and genes in the region between positions 3,507,000 and 4,107,000 bp on apple chromosome 2. The figure shows the region between 3,507,000 and 4,107,000 bp on chromosome 2 based on the ‘Golden Delicious’ v1.0p pseudohaplotype assembly [16]. This region contains resistance loci to apple scab, including *Rvi4/Vh4* and. *Rvi15/Vr2.* Tracks from outside to inside are: (1) region ideogram ticks show the position in megabases (Mb), (2) trimmed standard deviation of segmentation scores plotted as a histogram with a y-scale from 0 to 0.6 (red bars indicate regions designated as CNVRs, blue bars represent all other regions), (3) the location of resistance genes, and (4) the location of genes other than resistance genes (yellow bars represent the location of these genes)

**Fig. 7:**
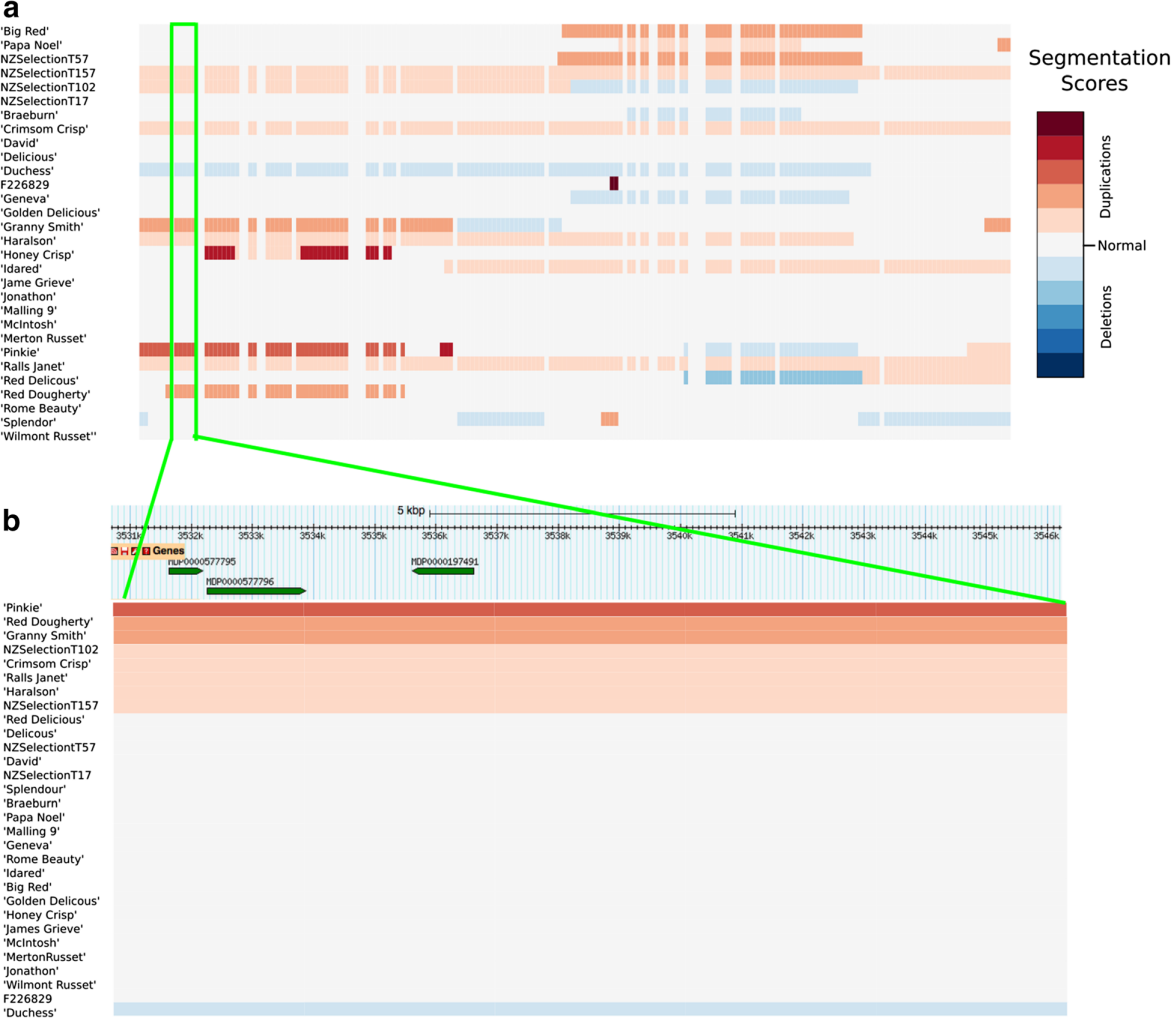
Heatmap representation of the segmentation scores of the region between positions 3,507,000 and 4,107,000 bp on apple chromosome 2. **a** Heatmap of segmentation scores with rows ordered in alphabetical order of accession name for the region chromosome 2 from 3,507,000 to 4,107,000 bp. The segmentation values are plotted for each window in the region; these values represent significant changes in read-depth signal. The green highlighted region shows one of the 19 CNVRs located within the region. **b** Heatmap of segmentation scores with rows ordered by segmentation score for the CNVR on chromosome 2 between positions 3,531,000 and 3,546,000 bp. The resistance gene model (MDP000057795) and the other gene models (MDP000057796 and MDP0000187491) are located above the heat map in their correct position relative to their location within the CNVR. The track above the heatmap displays the location of the gene models found within the CNV (extracted from the Rosaceae genome browser)

Numerous studies have focused on identifying genomic regions associated with horticultural traits in apple, using genetic linkage mapping and QTL analysis [see Troggio et al. for a review [59]. Three regions of the apple genome that contain putative CNVRs are of particular interest, as they co-locate with loci controlling traits related to biotic and abiotic responses in apple. Firstly, a cluster of 19 CNVRs between 3,507,000 bp and 4,107,000 bp on chromosome 2 contains multiple R genes models (Figs. 6 & 7) in a region where major loci conferring resistance to apple scab have been previously mapped. The *Rvi4/Vh4* and *Rvi15/Vr2* loci were originally mapped to apple linkage group 2 [60, 61] and the recent development of genetic markers closely linked to these loci enabled to estimate their physical location on the reference apple genome of ‘Golden Delicious’ [62], close to the detected cluster of 19 CNVRs. Secondly, a CNVR located at positions 3,506,106 and 3,509,841 bp on chromosome 11 co-locates with *Pl2*, a major gene conferring resistance to powdery mildew. *Pl2* was originally mapped using expressed sequence tags-based markers [62] and the newly developed markers for *Pl2* [51] enabled placement of this locus on the apple genome sequence in the same position as the CNVR. Finally, three CNVRs located between positions 1,404,000 and 1,671,000 bp on chromosome 9 co-locate with a QTL for budbreak date [50]. Future work needs to include validation of these CNVRs, to demonstrate linkages between the CNVRs on chromosomes 2, 11, and 9 for scab resistance, powdery mildew resistance and budbreak date, respectively. This validation can be carried out by re-sequencing, with higher depth of coverage, cultivars carrying the scab resistance, powdery mildew, and early budbreak date alleles, and comparing the resulting integer copy-number genotypes to those of cultivars not carrying the resistance. Using the higher coverage data, the exact breakpoint of an individual CNV can be determined, and in combination with the integer copy-number genotypes, will enable the design of genetic markers that will accurately quantify the copy number at these loci. In addition to extensive genetic validation, functional validation of CNVs should also be performed. This could be achieved by assessing the mRNA/protein expression of the candidate gene(s) (in an appropriate tissue) from accessions that have different copy-numbers. A significant positive correlation would suggest a functional link, where raised copy-number increased the expression of the candidate gene(s). We hypothesize that some of these CNVs may be causative for these loci and that these functional CNV markers will be more powerful than nearby SNP markers for application in marker-assisted breeding.

## Conclusion

We identified 876 CNVRs with an average size of 16.4 kb, comprising 3.5 % of the apple genome. These results represent the first catalogue and investigation of a previously unexplored form of genetic variation in a tree species. The CNVRs identified in this study are enriched in resistance (R) gene models and overlap with major gene loci of agricultural significance. Further investigation of apple CNV using higher coverage NGS data will enable integer-level copy-number assignment and break-point identification. This will facilitate the discovery of the causative CNV and improve the design of molecular markers that segregate with the trait. Ultimately, we believe that the focused investigation of CNV in the apple genome will lead to the genetic improvement of apple cultivars and a deeper understanding of the role CNV plays within the apple genome and other long-lived tree species.

## Additional files

Additional file 1:
**Sequencing and quality control result for the 30 apple varieties.** (XLSX 11 kb) Additional file 2:
**List of copy-number variable regions (CNVRs) identified in the apple genome.** (XLSX 41 kb) Additional file 3:
**Per chromosome summary of copy-number variable regions (CNVRs) in the apple genome.** (XLSX 10 kb) Additional file 4:
**List of all gene models found within copy-number variable regions (CNVRs) in the apple genome.** (XLSX 50 kb) Additional file 5:
**List of resistance (R) gene models located within copy-number variable regions (CNVRs) in the apple genome.** (XLSX 10 kb)
